# Monitoring Haematocrit in Paediatric Patients Receiving Testosterone Therapy in Arab Countries

**DOI:** 10.7759/cureus.17618

**Published:** 2021-08-31

**Authors:** Hussain Alsaffar, Abdelhadi Habeb, Rasha T Hamza, Asma Deeb

**Affiliations:** 1 Child Health Department - Paediatric Endocrinology Unit, Sultan Qaboos University Hospital, Muscat, OMN; 2 Paediatric Department, Prince Mohammed Bin Abdulaziz Hospital, Ministry of National Guard, Madinah, SAU; 3 Faculty of Medicine, Ain Shams University, Cairo, EGY; 4 Paediatric Endocrinology Department, Shaikh Shakhbout Medical City, Abu Dhabi, ARE

**Keywords:** haematocrit, erythrocytosis, testosterone, monitoring, arab, asped

## Abstract

Objectives

Testosterone is the main agent used to induce puberty in boys in Arab countries. It is recommended to monitor haematocrit before and during androgen replacement. However, data from single centre studies indicated that this recommendation is rarely practiced by paediatricians compared to adult physicians. The aim of this study is to evaluate the monitoring of haematocrit of patients on Testosterone therapy by paediatric endocrinologists practicing in Arab countries.

Methods

A cross-sectional study using an online survey that was sent to all members of the Arab Society for Paediatric Endocrinology and Diabetes (ASPED), who they practice in all Arab countries. The study was carried out between July and October 2019. Ethical approval was granted by ASPED council in May 2019 (MRE2019-02Q).

Results

One hundred four physicians responded to the survey from 17 countries. 81/104 (77.8%) answered the question about Testosterone monitoring (42 paediatric endocrinologists, 11 general paediatrician consultants with interest in endocrine, 16 specialists, four fellows and eight residents). Of the 81 responders 18 clinicians (22.2%) thought of monitoring the haematocrit; 15 (18.5%) thought no laboratory monitoring is needed at all.

Conclusion

The survey indicated that most paediatric endocrinologists in Arab countries do not monitor haematocrit in patients on testosterone replacement and majority are not aware that secondary erythrocytosis can result from androgen therapy. Raising the awareness on monitoring haematocrit during androgen replacement therapy is needed especially when reaching the adult dose.

## Introduction

In boys, puberty can be induced using the combination of long-term human choriogonadotropin (hCG) and follicular stimulating hormone therapy in cases of secondary hypogonadism [1,2]. Whereas, testosterone is the main used agent for inducing puberty in primary gonadal insufficiency or constitutional delay of growth and puberty. It has shown a great role in promoting linear growth and developing secondary sexual characteristics. Bone health and muscle mass are also influenced by testosterone [3]. Testosterone therapy does not affect the success of using gonadotropins in the future when fertility is desired. Testosterone is available in different formulations; Testosterone enantate or propionate or alternatively Sustanon® are given intramuscularly and they are preferred for replacement therapy due to their longer duration. Other formulations and preparations include oral testosterone undecanoate, subcutaneous testosterone injections, transdermal testosterone and topical gel. Sustanon® (which consists of a mixture of testosterone esters) is the most widely available preparation in MENA (Middle East and North Africa) region. Other preparations are infrequently available [4]. Most of the clinicians who responded to Arab Society of Paediatric Endocrinology and Diabetes’s (ASPED) survey are using 50 mg/month as a starting dose of Testosterone for puberty induction in males [5] and the dose can be escalated depends on the response.

Since testosterone has an erythropoietic effect, that could lead to polycythaemia, or erythrocytosis [6] detected by elevated haematocrit [7-9], the Endocrine Society recommends monitoring testosterone and haematocrit levels in men, three to six months after initiating the treatment, at 12 months and then annually [10], whilst the Endocrine Society of Australia recommends haematology profile three months after starting Testosterone therapy and then annually [11]. The risk of polycythaemia is even higher with intramuscular route compared to other routes of Testosterone administration [12,13]. However, it is still complicating other formulations such as subcutaneous Testosterone, with a positive correlation between the risk and duration of replacement therapy [14,15]. Polycythaemia secondary to Testosterone therapy can cause symptoms of hyperviscosity mainly in adults such as paraesthesia, headache, fatigue, and blurred vision [16]. Other reported, unexpected, and serious complications include branch retinal artery occlusion [17], cerebral infarctions [18], sensorineural hearing loss [19] and high prevalence of obstructive sleep apnoea [20]. However, testosterone therapy has not been associated with an increased risk of deep venous thrombosis [21]. Patients with elevated haematocrit may require therapeutic phlebotomy “venesection” [22] especially if packed cell volume is more than 54% [16].

For children and adolescents, there is no clear statement from Paediatric Endocrine Societies regarding the monitoring of Testosterone use, perhaps because of the short duration of its use for puberty induction in adolescents or because the fact that the risk of developing cardiovascular diseases secondary to polycythaemia and viscosity is much lower in this age group compared to adults. However, British National Formulary for Children (BNF-C) [23] and Electronic Medicines Compendium [24] recommend haematocrit monitoring in paediatric age group without further specification. Overall, there is a paucity of studies describing the safety and monitoring of the testosterone therapy’s adverse events when used in paediatric age groups. Lucas-Herald et al [25] from Glasgow wrote about their experience of using Testosterone in treating boys with hypogonadism, highlighting that generally the monitoring of testosterone safety in paediatrics is poor and not standardised, only 6% of their patients had their haematocrit checked during Testosterone therapy. On the other side, Vogiatzi et al suggested monitoring for polycythaemia in adolescents only when reaching adult Testosterone dose as the response of haemoglobin to Testosterone administration is dose-dependent [26]. Our study aims to explore the experience and awareness of clinicians practicing paediatric endocrinology in Arab countries about monitoring haematocrit of their patients who are receiving testosterone therapy. 

## Materials and methods

Study design

An online survey using a commercial website (Survey Monkey, USA) about puberty induction was sent out to practicing physicians in Arab countries according to the Arab Society of Paediatric Endocrinology and Diabetes (ASPED) database. The study was approved by the ASPED council for which ethical approval was granted. An invitation link was emailed, and responses were collected between July and October 2019. Respondents had the right to decline participation and data were collected anonymously. 

The questionnaire

After the demographic questions, clinicians were asked about monitoring the safety of androgens when used for induction of puberty and thereafter as a replacement therapy. Different options of relevant and irrelevant laboratory investigations were provided in the survey and responders were asked to select their choice of investigations. The survey’s language was English, the official language of communication between paediatric endocrinologists in the region.

Statistical analysis

Data got summarised anonymously using descriptive statistics. p-value was calculated using z-score calculator from social science statistics [27], p-value less than 0.05 was considered statistically significant.

## Results

**Figure 1:**
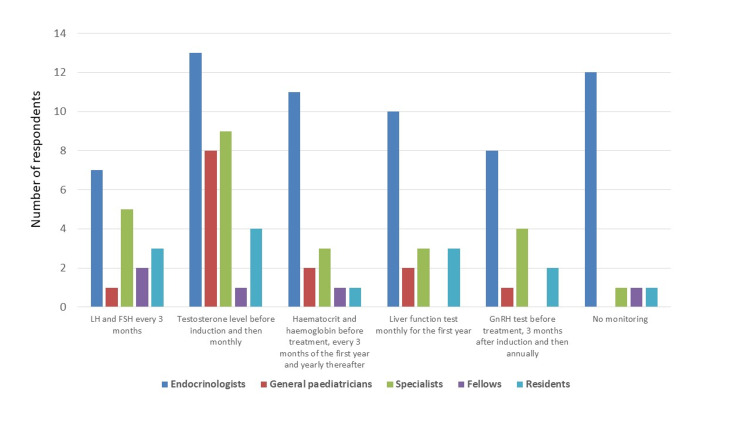
Responses of clinicians to the question about the required laboratory investigations for monitoring when giving androgens for long term (81 clinicians responded to this question, more than one answer can be selected by each respondent).

In total, 104 physicians from 17 countries (Algeria, Bahrain, Egypt, Iraq, Jordan, Kuwait, Lebanon, Libya, Morocco, Oman, Palestine, Qatar, Saudi Arabia, Sudan, Tunisia, United Arab Emirates and Yemen) agreed to participate. Ninety-three would use testosterone to induce puberty. Of these, 80.6% (75/93) prefer intramuscular preparation for induction and 66/93 (70.9%) use it for maintenance as a replacement. Out of the 104 participants, 81 (77.9%) responded to the raised question about safety monitoring of testosterone (42 paediatric endocrinologists, 11 general paediatrician consultants with special interest in paediatric endocrinology, 16 specialists, four fellows and eight residents). Only 18/81 (22.2%) of the respondents to this question, thought of monitoring the haematocrit. Of them, 11 endocrinologists, by which, only 26% (11/42) of the total 42 responding endocrinologists to this question, are monitoring the haematocrit. 15/81 respondents from seven countries (18.5%) thought no haematological or biochemical monitoring are needed. 18/81 clinicians (10 endocrinologist consultants, two general paediatricians, three specialists and three residents) selected the liver function test to be checked monthly for the first year. 35/81 selected to monitor testosterone level before induction and then every three months. 18 selected LH and FSH on three monthly basis and 16 others selected GnRH to be performed before induction of puberty, three months later and annually afterwards. Details are shown in Figure 1.

Table 1 summarises the comparison of the responses received from endocrinologists versus others (general paediatrician with interest in endocrine, specialists, residents, and fellows). “No monitoring” option was opted more by the endocrinologists’ group with a statistical significance (p: 0.007) whereas, the others’ group chose to monitor testosterone levels monthly, more than the endocrinologists (p: 0.01). 

**Table 1:** Comparison between answers of endocrinologists versus others (general paediatricians with interest in endocrine, specialists, fellows, and residents). Respondents were able to select more than one option. LH: luteinizing hormone; FSH: follicle-stimulating hormone.

Test	Endocrinologists (n = 42)	Others (n = 39)	Total responses	p-value
LH and FSH every three months	7	11	18	0.1
Testosterone level before induction and then monthly	13	22	35	0.01
Haematocrit and haemoglobin before treatment, every three months of the first year and yearly thereafter	11	7	18	0.18
Liver function test monthly for the first year	10	8	18	0.36
GnRH test before treatment, three months after induction and then annually	8	7	15	0.44
No monitoring	12	3	15	0.007

## Discussion

Choosing the medicine and formulation for induction of puberty in males is usually depending on the availability, cost, convenience, and familiarity [28]. Testosterone is very widely used worldwide, and it is the most used agent in ASPED countries for puberty induction [5]. The monitoring of Testosterone therapy needs standardisation in paediatrics [25]. Stancampiano et al [29] suggested the monitoring of Testosterone therapy should be determined by the underlying pathology or the intension duration of Testosterone replacement whether it is temporary as in Constitutional Delay of Growth and Puberty (CDGP) or permanent as in hypogonadism. Laboratory investigations including full blood count (FBC) and LFT were recommended at baseline whether temporary or permanent plan of testosterone replacement. Furthermore, in patients with hypogonadism, it was recommended monitoring the FBC (Haemoglobin and haematocrit) and total testosterone at 3, 6, 12 months, then annually for FBC and 6monthly for testosterone. Lipid profile, bone age and bone mineral density (BMD) to be checked at baseline, 12 months and then every one to two years [29].

In this survey, the question focused on the monitoring the safety and pharmacokinetics of Testosterone therapy in boys with hypogonadism whom requiring testosterone for longer than just induction over a period of three to six months. We have observed variation of clinical practice among clinicians in ASPED countries, regarding the selection of the appropriate laboratory investigations for monitoring. Comparing our results with previous studies, haematocrit considered to be checked by 18/81 (22.2%) of our respondents. Whereas a retrospective study at Boston Children’s Hospital between 2006 and 2013, revealed that haematocrit was checked in 41/59 (69.5%) adolescents and young adults [30]. Another retrospective study from Royal Hospital for Children in Glasgow, between 2012 and 2017 showed haematocrit was checked in 6/46 (13%) (Table 2) [24]. There is a great potential to improve this practice, considering the other recommended investigations such as bone age and DEXA scan for BMD. Our survey focused only on the laboratory investigations, which gave the study some limitations.

**Table 2:** Summary of previous relevant studies compared to ASPED clinicians’ practice. ASPED: Arab Society for Paediatric Endocrinology and Diabetes.

Study	Population	(n)	Hct monitoring during Testosterone therapy
Nahata et al (2015) [30]	Adolescents and young adults aged ≥ 16 years	59	(41/59) 69%
Lucas-Herald (2018) [25]	Adolescents (12.1 to 17.7 years)	46	(6/46) 13%
Our study	Clinicians were asked	81	(18/81) 22.2%

To our knowledge erythrocytosis was not reported in adolescents as a complication of Testosterone therapy in our region, but whether that is because of lack of awareness, hence under reporting, or because of the rarity of this complication in youngsters. Further studies are deemed necessary, but first we will aim to raise the awareness of clinicians in our region about the recommended scheme of investigations [29], followed by a prospective study to give a better insight. 

Other options for selection that were provided in the survey included FSH and LH monitoring. This could be justifiable in patients with primary gonadal failure receiving replacement therapy to examine the effect of androgen on suppressing the gonadotrophs, but not for hypogonadotrophic hypogonadism, where it is only recommended as a baseline investigation

Liver function was suggested to be monitored six-monthly, to screen for transaminitis [31]. However, the Endocrine Society did not recommend monitoring liver function test in its most recent guideline. In fact, it was considered unnecessary investigations for men taking intramuscular injections or transdermal preparations as these forms are not associated with liver dysfunction [32]. Rather, it is only suggested to be checked at baseline, 6-12 months and as warranted clinically for patients using oral alkylated agents [33] which we do not use frequently in our region.

Measurement of testosterone level is suggested 6-12 weeks after initiation of the replacement therapy, and for those patients receiving injectable forms (testosterone enanthate or cypionate) to have an earlier measurement of serum testosterone at one to two weeks after commencement of therapy [34]. But we were not expecting 35 clinicians to consider a monthly testosterone level measurement!

Lastly, GnRH test is neither justifiable nor recommended for monitoring patients on testosterone. In fact, we are surprised that 16/81 (19.8%) would perform this test 3 months after starting treatment and yearly thereafter. This could be just a waste of resources, creating unnecessary anxiety and patients’ discomfort. We could not see any justification for conducting GnRH for patients on Testosterone therapy, hence the surprise that we had when it was selected. 

## Conclusions

The authors would recommend standardisation of testosterone therapy monitoring in ASPED countries. Haematocrit and total testosterone level checking at baseline, three-monthly for the first year, followed by annual FBC and six-monthly total testosterone level. When there is no evidence of transaminitis at the baseline, liver function tests to be monitored only for patients on oral alkylated agents. Lipid profile, DEXA scan and bone age to be done at baseline and 1-2 yearly thereafter. By which, this will ensure the achievement of patient comfort, as well as time and resources savings by minimising the unnecessary investigations and over medicalisation. Long-term prospective study is recommended in our region to evaluate the prevalence of erythrocytosis in people younger than 20 years of age. 

## References

[1] Barrio R, de Luis D, Alonso M, Lamas A, Moreno JC (1999). Induction of puberty with human chorionic gonadotropin and follicle-stimulating hormone in adolescent males with hypogonadotropic hypogonadism. Fertil Steril.

[2] Zacharin M, Sabin MA, Nair VV, Dabadghao P (2012). Addition of recombinant follicle-stimulating hormone to human chorionic gonadotropin treatment in adolescents and young adults with hypogonadotropic hypogonadism promotes normal testicular growth and may promote early spermatogenesis. Fertil Steril.

[3] Yin A, Swerdloff R (2010). Treating hypogonadism in younger males. Expert Opin Pharmacother.

[4] Deeb A, AlSaffar H, Hamza RT, Habeb A (2020). Availability of medications used for puberty induction and maintenance in adolescents with hypogonadism in the Arab region. bioRxiv.

[5] Hamza RT, Deeb A, Al Saffar H, Alani SH, Habeb A (2020). Timing and regimen of puberty induction in children with hypogonadism: a survey on the practice in Arab countries. J Pediatr Endocrinol Metab.

[6] Ohlander SJ, Varghese B, Pastuszak AW (2018). Erythrocytosis Following Testosterone Therapy. Sex Med Rev.

[7] Sehn E, Mozak C, Yuksel N, Sadowski CA (2019). An analysis of online content related to testosterone supplementation. Aging Male.

[8] Winters SJ (2016). Monitoring testosterone levels in testosterone-treated men. Curr Med Res Opin.

[9] Dobs AS, Wayne-Meikle A, Arver S, Sanders SW, Caramelli KE, Mazer NA (1999). Pharmacokinetics, efficacy, and safety of a permeation-enhanced testosterone transdermal system in comparison with bi-weekly injections of testosterone enanthate for the treatment of hypogonadal men. J Clin Endocrinol Metab.

[10] Bhasin S, Brito JP, Cunningham GR (2018). Testosterone therapy in men with hypogonadism: an endocrine society clinical practice guideline. J Clin Endocrinol Metab.

[11] Yeap BB, Grossmann M, McLachlan RI (2016). Endocrine Society of Australia position statement on male hypogonadism (part 2): treatment and therapeutic considerations. Med J Aust.

[12] Nolan BJ, Leemaqz SY, Ooi O (2021). Prevalence of polycythaemia with different formulations of testosterone therapy in transmasculine individuals. Intern Med J.

[13] Jick SS, Hagberg KW (2013). The risk of adverse outcomes in association with use of testosterone products: a cohort study using the UK-based general practice research database. Br J Clin Pharmacol.

[14] Rotker KL, Alavian M, Nelson B, Baird GL, Miner MM, Sigman M, Hwang K (2018). Association of subcutaneous testosterone pellet therapy with developing secondary polycythemia. Asian J Androl.

[15] Kaminetsky JC, McCullough A, Hwang K, Jaffe JS, Wang C, Swerdloff RS (2019). A 52-week study of dose adjusted subcutaneous testosterone enanthate in oil self-administered via disposable auto-injector. J Urol.

[16] Cervi A, Balitsky AK (2017). Testosterone use causing erythrocytosis. CMAJ.

[17] Choy DM, Voon LW, Teoh SC (2019). Unusual cause of branch retinal artery occlusion: Polycythemia in a transgender man from unregulated testosterone use. Retin Cases Brief Rep.

[18] Low MS, Vilcassim S, Fedele P, Grigoriadis G (2016). Anabolic androgenic steroids, an easily forgotten cause of polycythaemia and cerebral infarction. Intern Med J.

[19] Tikka T, Mistry N, Janjua A (2016). Acute unilateral sensorineural hearing loss associated with anabolic steroids and polycythaemia: case report. J Laryngol Otol.

[20] Lundy SD, Parekh NV, Shoskes DA (2020). Obstructive sleep apnea is associated with polycythemia in hypogonadal men on testosterone replacement therapy. J Sex Med.

[21] Kavoussi PK, Machen GL, Wenzel JL, Ellis AM, Kavoussi M, Kavoussi KM, Kavoussi SK (2019). Medical treatments for hypogonadism do not significantly increase the risk of deep vein thrombosis over general population risk. Urology.

[22] Van Buren NL, Hove AJ, French TA, Gorlin JB (2020). Therapeutic phlebotomy for testosterone-induced polycythemia. Am J Clin Pathol.

[23] (2020). BNFC - Medicines Complete. BNF.

[24] (2020). EMC - Medicines. EMC.

[25] Lucas-Herald AK, Mason E, Beaumont P, Mason A, Shaikh MG, Wong SC, Ahmed SF (2018). Single-centre experience of testosterone therapy for boys with hypogonadism. Horm Res Paediatr.

[26] Vogiatzi M, Tursi JP, Jaffe JS, Hobson S, Rogol AD (2021). Testosterone use in adolescent males: current practice and unmet needs. J Endocr Soc.

[27] (2020). socscistatistics. https://www.socscistatistics.com/tests/ztest/default2.aspx..

[28] Conway AJ, Handelsman DJ, Lording DW, Stuckey B, Zajac JD (2000). Use, misuse and abuse of androgens. The Endocrine Society of Australia consensus guidelines for androgen prescribing. Med J Aust.

[29] Stancampiano MR, Lucas-Herald AK, Russo G, Rogol AD, Ahmed SF (2019). Testosterone therapy in adolescent boys: the need for a structured approach. Horm Res Paediatr.

[30] Nahata L, Yu RN, Bhasin S, Cohen LE (2015). Management of testosterone therapy in adolescents and young men with hypogonadism: are we following adult clinical practice guidelines?. J Pediatr Endocrinol Metab.

[31] Collins-Bride G, Saxe J (2013). Clinical Guidelines for Advanced Practice Nursing: An Interdisciplinary Approach. https://books.google.com.om/books?hl=en&lr=&id=p7W1jATjPOQC&oi=fnd&pg=PR1&dq=Clinical+Guidelines+for+Advanced+Practice+Nursing:+An+Interdisciplinary+Approach.+Second.+USA:+Jones+and+Bartlett+Learning+Publications&ots=5QNt3pq3c0&sig=fzv6N2KRE-AOIIQ8s3FCYoNb8BU&redir_esc=y#v=onepage&q=Clinical%20Guidelines%20for%20Advanced%20Practice%20Nursing%3A%20An%20Interdisciplinary%20Approach.%20Second.%20USA%3A%20Jones%20and%20Bartlett%20Learning%20Publications&f=false.

[32] Rhoden EL, Morgentaler A (2004). Risks of testosterone-replacement therapy and recommendations for monitoring. N Engl J Med.

[33] Seftel A (2007). Testosterone replacement therapy for male hypogonadism: part III. Pharmacologic and clinical profiles, monitoring, safety issues, and potential future agents. Int J Impot Res.

[34] Carnegie C (2004). Diagnosis of hypogonadism: clinical assessments and laboratory tests. Rev Urol.

